# Multiplex HPV RNA in situ hybridization/p16 immunohistochemistry: a novel approach to detect papillomavirus in HPV-related cancers. A novel multiplex ISH/IHC assay to detect HPV

**DOI:** 10.1186/s13027-020-00310-x

**Published:** 2020-07-14

**Authors:** Federica Zito Marino, Andrea Ronchi, Marianna Stilo, Immacolata Cozzolino, Elvira La Mantia, Nicola Colacurci, Giuseppe Colella, Renato Franco

**Affiliations:** 1 Pathology Unit, Department of Mental and Physical Health and Preventive Medicine, University of Campania “L. Vanvitelli”, 80138 Naples, Italy; 2 Department of Woman, Child and General and Specialized Surgery, Obstetrics and Gynecology Unit, University of Campania “L. Vanvitelli”, 80138 Naples, Italy; 3 Maxillofacial Surgery Unit, Department of Medical, Surgical and Dental Speciality, University of Campania “L. Vanvitelli”, 80138 Naples, Italy

**Keywords:** ISH, HPV, p16, Cervical Cancer and oral cancer

## Abstract

**Background:**

High-risk human papillomavirus (HR-HPV) is notoriously associated with tumor progression in a broad spectrum of malignancies. Detection of HR-HPV is clinically important in the management of HPV-related carcinomas, particularly in cervical cancer and oropharyngeal squamous cell carcinoma (OPSCC). Several methods for HPV detection are currently available including Polymerase chain reaction (PCR)-based techniques, DNA in situ hybridization (ISH), RNA ISH, and p16 immunohistochemistry (IHC). Currently, the guidelines for HPV detection in cervical carcinoma are available, while no clear consensus has not yet been reached on the gold standard for HPV testing in OPSCC. Multimodality testing could help to reliably identify patients with transcriptionally active high-risk HPV-positive.

**Methods:**

We propose a multiplex approach carrying out HPV RNA ISH and p16 IHC on the same slide to detect simultaneously HPV E6/E7 transcripts and p16INK4a overexpression. We tested this assay in two different series one of the cervical cancers with p16-positive, as control, and the other of oropharyngeal squamous cell carcinomas with blind p16 status.

**Results:**

The multiplex HPV RNA ISH /p16 IHC results in the series both of the cervical cancers and the oral-oropharyngeal cancers were fully concordant with the previous results achieved through the classic p16 IHC and HPV RNA scope carried out on two different slides.

**Conclusions:**

Our results suggesting several advantages of this technical approach, namely an easy interpretation fully in the light field, the feasibility in formalin-fixed paraffin-embedded tissue sections, complete automation and a potential wide spreadable for routine testing in several clinical laboratories.

## Background

Human papillomavirus (HPV) is non-enveloped icosahedral, circular, dsDNA virus that can infect cutaneous and mucosal epithelia. Approximately 200 different HPV genotypes have been identified responsible of a broad spectrum of the clinical profiles, from benign lesions to HPV-related carcinomas [1, 2]. High-risk HPV (HR-HPV), most commonly types 16 and 18, have a great oncogenic potential, leading to HPV-related carcinomas. HPV has been reported to be an etiological agent for approximately 5% of all the human cancers, particularly the cervical, oropharyngeal, vaginal, vulval, penile cancers and lung cancer [3, 4]. The HR-HPV persistent infection is the cause of up to 85% of cervical cancer in women, due generally to genotypes 16 and 18 [5, 6].

The US Preventative Services Task Force (USPSTF) recommends that women aged 30 to 65 years should be screened with cytology and HPV testing (co-testing) every 5 years [7]. Instead, the European guidelines for quality assurance in cervical cancer screening recommend to test for HPV alone in women aged 30–65 every five years [8].

In the last decade, HR-HPV has revolutionized head and neck oncology, since the HPV-positive oropharyngeal squamous cell carcinoma (OPSCC) represents a unique cancer type with distinct clinicopathologic features and favorable prognosis. Thus HPV-OPSCC is now a well-recognized tumoral entity in the field of head and neck oncology, with an increasing incidence [9, 10]. Several methods for HPV detection are currently available including Polymerase chain reaction (PCR)-based techniques, DNA in situ hybridization (ISH), RNA ISH and p16 immunohistochemisty (IHC) [11]. The PCR-based detection of the HPV DNA is exclusively able to quantify viral load, however it cannot distinguish the HPV transcriptionally-active infections from those defined as “passenger” HPV [12–14]. The quantitative reverse transcriptase PCR to detect HPV E6/E7 mRNA would seem to be an ideal HPV testing method, since it reveals that HPV is not merely present, but is transcriptionally active [13, 14]. The Food and Drug Administration (FDA) approved the PCR to detect E6/E7 mRNA as the “gold standard” for the detection and typing of HPV [15]. Unfortunately, this assay can be performed exclusively in fresh-frozen tumor tissue, leading its limited use in the clinical practice. RNA in situ hybridization for detecting the HPV E6/E7 mRNA transcripts represents an advance in HPV testing, because of the ability to detect the virus in its transcriptionally-active status also in formalin-fixed paraffin-embedded tumor tissues (FFPE) [11, 14]. RNA ISH is incredibly sensitive, being near to 100%, however, the assay showed a specificity approximately of 90% [16]. Another technique used in the clinical practice to HPV testing is the immunohistochemical detection of p16INK4a overexpression, used as a surrogate biomarker of viral activity mainly in cervical and oropharyngeal cancers [8, 14, 17]. p16 IHC represents a surrogate for transcriptionally-active HR HPV since HPV viral oncoprotein E7 signaling induces strong overexpression of the cellular protein p16INK4a in HPV-transformed cells [18].

p16 IHC represents a method relatively inexpensive, which is highly sensitive for transcriptionally active HR HPV [13, 19, 20]. Although a good concordance between RNA ISH and p16 IHC has been reported, there is no exclusive biologic link between p16 over-expression, HPV infection, and carcinogenesis [17].

In this context, multimodality testing could help an accurate detection of HPV. We developed a multiplex approach carrying out HPV RNA ISH and p16IHC on the same slide to detect simultaneously HPV E6/E7 transcripts and p16INK4a overexpression. We validated this approach in two different series, one of the cervical cancers and the other of OPSCC .

## Methods

### Specimens

A series of 17 cervical cancers with p16-positive IHC and a series of 38 oral-oropharyngeal cancers with blind p16 status were included in our study. All cases were collected in our records at the University of Campania “L. Vanvitelli”. The series included surgical samples or wide biopsies. Sections of 4 μm thickness from each block with a mean of 3 blocks per tumor) were stained with hematoxylin-eosin. All cases were reviewed according to the histopathological classification.

### p16 immunohistochemistry

p16 IHC was carried out with a proprietary kit CIN tec Histology; MTM laboratories AG) using the clone E6H4 on a Ventana Benchmark autostainer (Ventana Medical Systems, Tucson, AZ, USA) for the detection of p16INK4a antigen. A tonsil squamous cell carcinoma with high p16 expression was used as a positive control. The primary antibody was omitted from negative controls. The new guidelines proposed by the College of American Pathologists (CAP), for HPV testing in head and neck carcinomas in routine clinical practice proposed the interpretation of p16 IHC as follows: tumors with lack of staining or < 70% of nuclear and cytoplasmic staining are classified as HPV negative, while tumors with ≥70% of nuclear and cytoplasmic staining as HPV positive [10]. According to the new CAP guidelines we evaluated as positive p16 IHC expression tumors with staining ≥70% nuclear and cytoplasmic staining, however in our analysis we identified also other subgroups with different p16 IHC staining. Finally, we interpreted p16 IHC as follows: p16 high expression: tumors with staining ≥70% nuclear and cytoplasmic staining; p16 moderate expression: tumors with staining 30–70% nuclear and cytoplasmic staining; p16 low expression: tumors with staining 10–30% nuclear and cytoplasmic staining; p16 negative: tumors with staining 1–10% nuclear and cytoplasmic staining. The slides were independently evaluated by three separate observers (**FZM, AR and RF)**.

### Automated HPV RNA in situ hybridization

Sections 4 *μ* mof each case are used to perform HPV RNA ISH test. Detection of high-risk-HPV E6/E7 mRNA was performed using Ready-to-use reagents from RNAscope 2.5 LS Reagent Kit-BROWN and the HPV-HR18 probe cocktail (Advanced Cell Diagnostics) that were loaded onto the Leica Biosystems’ BOND RX Research Advanced Staining System according to the user manual (Doc. No. 322100-USM). The slides were independently evaluated by three separate observers (**FZM, AR and RF)**. Ubiquitin C and dapB were used as positive and negative controls, respectively. A positive HPV ISH test result was defined as positive if any of the malignant cells showed brown punctate dot-like nuclear and/or cytoplasmatic positivity [21, 22].

### Multiplex HPV RNA in situ hybridization ISH/p16 immunohistochemistry

All steps are performed on the Leica BOND RX, automated system (Leica Microsystems,Bannockburn, IL). We tested different technical conditions.

In particularly, we have tested different dilutions of the antibody for the detection of p16INK4a antigen; different protocols namely first RNA ISH and then p16IHC or the opposite sequence; different colorimetric approaches including detection for HPV mRNA in DAB and p16 staining in Fast Red or conversely.

**Fig. 1:**
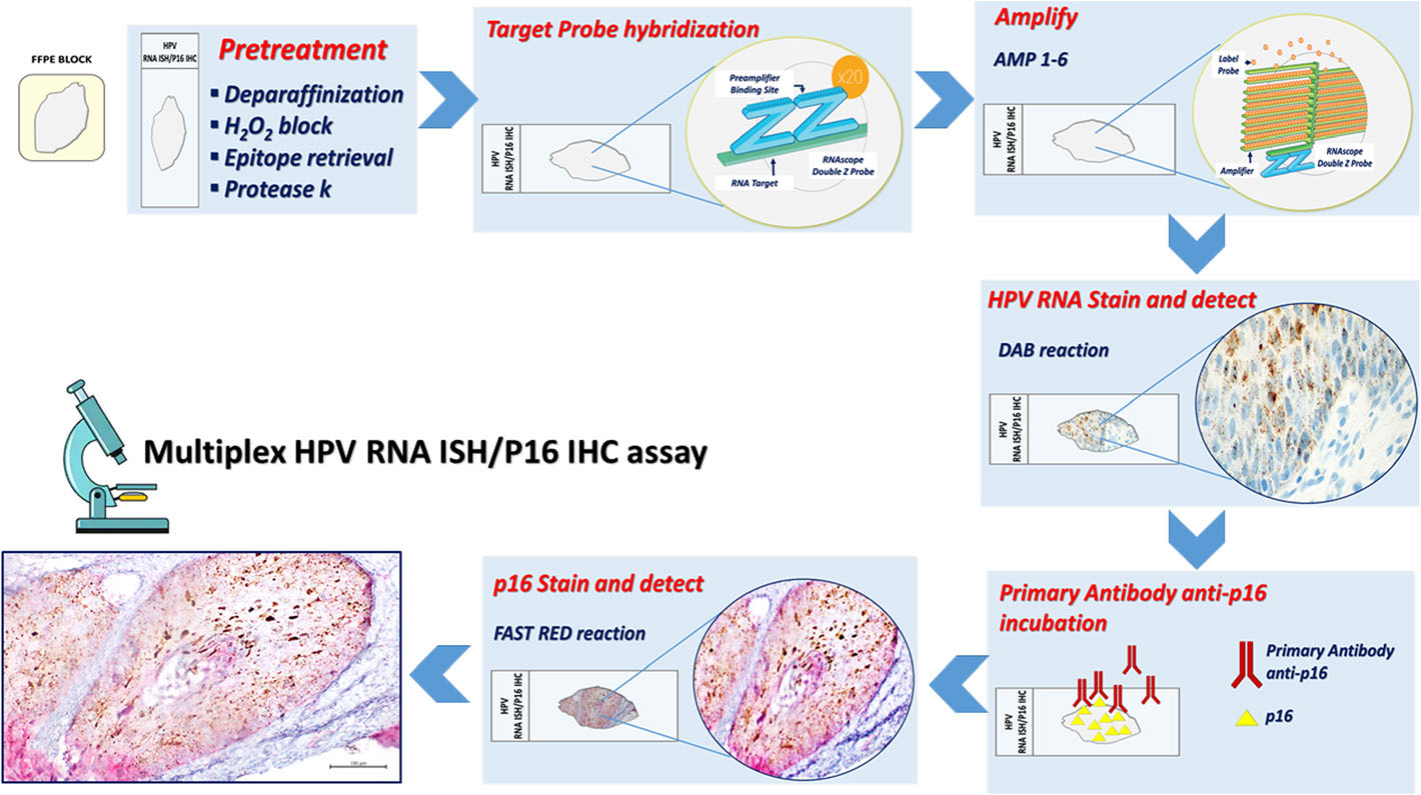
Schematic workflow of multiplex HPV RNA ISH/p16 IHC assay

Finally, our results showed that the sequential staining first RNA ISH in DAB and then p16 IHC staining in Fast Red represents the best technical approach (Fig. 1).

The protocol utilizes the Diaminobenzidine (DAB) chromogen of the Bond Polymer Refine kit to staining HPV E6/E7 mRNA, the Fast Red chromogen of the Bond Polymer Red Refine kit to staining p16 and hematoxylin to counterstain.

Detection of high-risk-HPV E6/E7 mRNA was performed using ready-to-use reagents from RNAscope® 2.5 LS Reagent Kit-BROWN and the HPV-HR18 probe cocktail (Advanced Cell Diagnostics) that were loaded onto the Leica Biosystems’ BOND RX Research Advanced Staining System according to the user manual (Doc. No. 322100-USM). The target-specific probes for the E6 and E7 genes of 18 HR-HPV genotypes HPV (16,18, 26,31, 33, 35, 39, 45, 51, 52, 53, 56, 58, 59, 66, 68, 73 and 82). The Ubiquitin C a constitutively expressed endogenous gene was used as positive control to assess the presence adequate RNA quality and avoid a false-negative result. The dapB test was used as negative control to assess non-specific staining, for a comparison in the cases with negative or weakly stained HPV staining.

In brief, 4 μm sections were baked and deparaffinized on the instrument, followed by epitope retrieval using Leica Epitope Retrieval Buffer 2 at 95 °C or at 88 °C for 15 min and protease treatment 15 min at 40 °C. Probe hybridization, signal amplification trough different AMP reagent AMP 1–6) and colorimetric detection were subsequently performed. Several washes were performed, subsequently the ready-to-use primary antibody clone E6H4 for the detection of p16INK4a antigen was incubated and colorimetric detection was performed. Finally, a hematoxylin staining was carried out.

When the run is completed and the slide trays are removed, the covertiles are carefully lifted upward by the neck to remove. The slides are dehydrated through 2 changes each of 70, 95, and 100% alcohol and 2 changes of xylene, before coverslipping. A schematic of multiplex HPV RNA ISH/p16 IHC assay workflow is presented in Fig. 1.

A positive HPV ISH test result was defined as positive if any of the malignant cells showed brown punctate dot-like nuclear and/or cytoplasmatic positivity. p16 IHC was positive if nuclear and cytoplasmic red staining was observed according to the above score. The slides were independently analyzed by three separate observers (**FZM, AR and RF)** evaluating simultaneously HPV mRNA and p16 expression.

### Statistical analysis

Fisher’s exact test was used to assess the correlation between the results obtained from multiplex HPV RNA ISH /p16 IHC and the achieved through the classic p16 IHC and HPV RNA scope carried out on two different slides. Statistical significance was set at *p* = 0.05. Data analysis and summarization were conducted using SPSS 20.0 for Mac (SPSS Inc., Chicago, Ill).

## Results

### Results in the series of cervical cancers

**Fig. 2:**
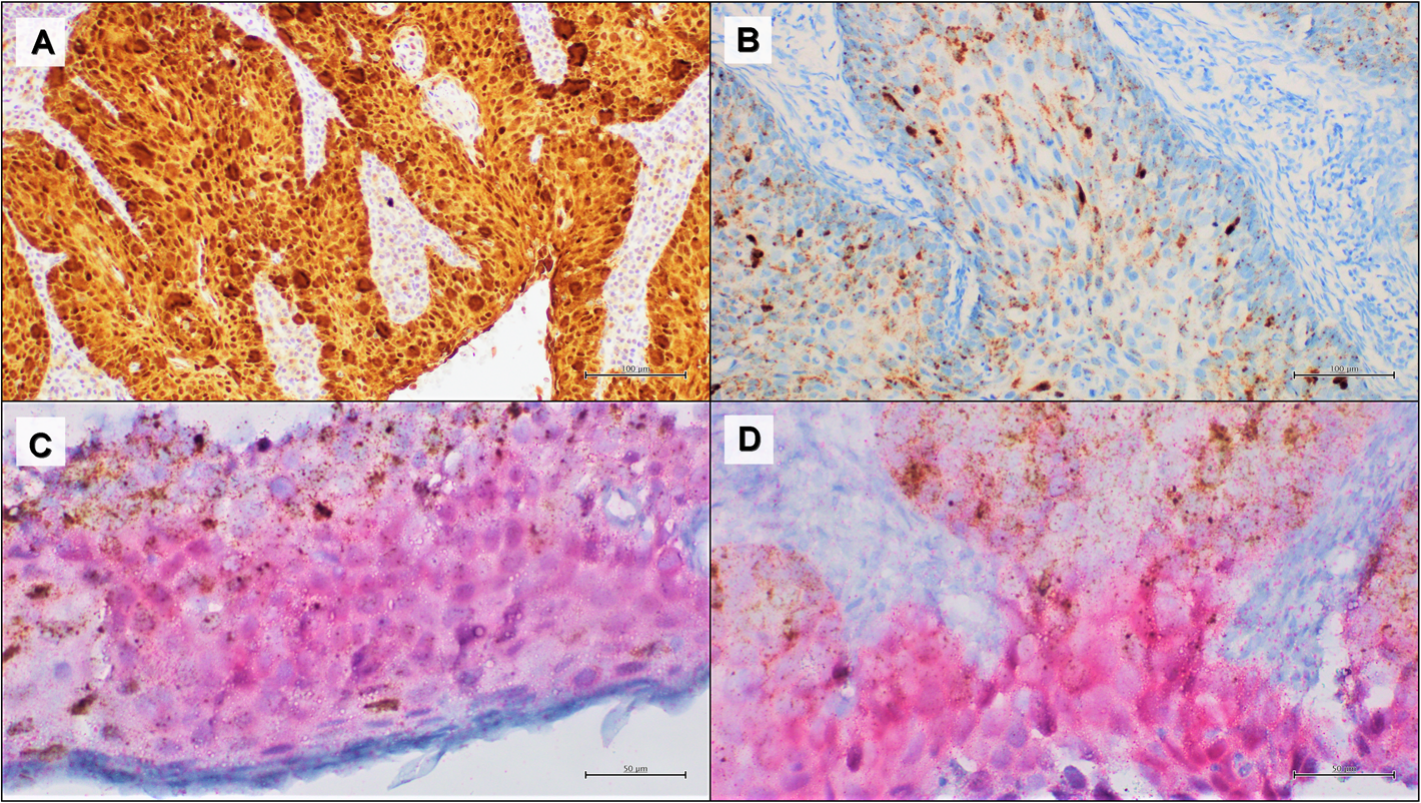
Representative results of a cervical cancer with high p16 and HPV RNA expression. **a** positive p16 IHC, DAB staining (100x); **b** positive HPV RNA in situ hybridization, DAB staining (100x); **c-d** multiplex HPV RNA ISH/p16 IHC: positive p16 IHC Fast Red staining and positive HPV RNA ISH DAB staining (50x)

The cervical cancers selected from our archive on the basis of p16-positive IHC status were tested for RNAscope HPV-test. All 17 cervical cancers p16-positive showed RNAscope HPV-test positive results (100% concordance). The multiplex HPV RNA ISH/p16 IHC analysis showed a complete correspondence with the data obtained using the p16 IHC test and RNAscope HPV-test carried out separately on two different slides (Fig. 2).

### Results in the series of oral-oropharyngeal cancers

Firstly, the p16 IHC and the RNAscope HPV-test were carried out separately on two different slides for each case. The p16 immunohistochemical expression was found in 7 (18.4%) of 38 cases of oral-oropharyngeal cancers, particularly 3 cases p16-positive (7.9%) showed high expression, 2 cases (5.3%) moderate expression and 2 cases (5.3%) low expression. The HPV mRNA was found in 3 (7.9%) out of 38 cases, particularly 1 case (2.6%) showed high positivity and 2 cases (5.3%) low positivity, all other cases were negative.

Overall, only two cases (5.3%) out of 38 showed concurrently p16 and HPV RNA expression, particularly one showing high expression and other low.

Overall, 6 (15.8%) out of 38 showed no corresponding test results between IHC and ISH. The p16 IHC analysis yielded positive results for five cases where there was an absence of detectable HPV mRNA by ISH. One case HPV RNA ISH positive was p16 IHC negative.

All 38 cases of oral-oropharyngeal cancers were tested with the multiplex HPV RNA ISH/p16 IHC and the results were compared with those obtained with the p16 IHC and HPV RNA test performed on two different samples.

**Fig. 3:**
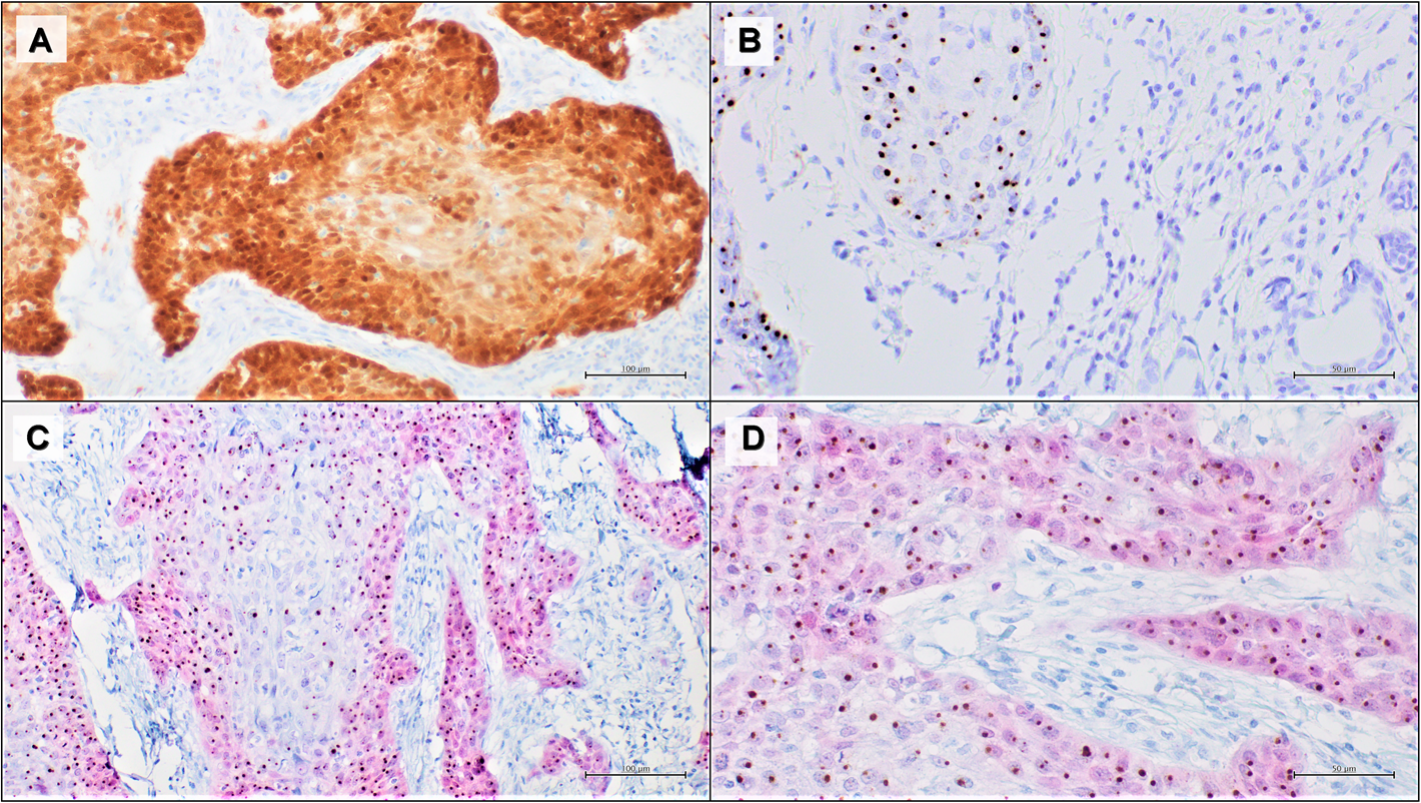
Representative results of an oropharyngeal squamous cell carcinoma with high p16 and HPV RNA expression. **a** positive p16 IHC, DAB staining (100x); **b** positive HPV RNA in situ hybridization, DAB staining (50x); **c** multiplex HPV RNA ISH/p16 IHC: positive p16 IHC Fast Red staining and positive HPV RNA ISH DAB staining (100x); **d** multiplex HPV RNA ISH/p16 IHC: positive p16 IHC Fast Red staining and positive HPV RNA ISH DAB staining (50x)

**Fig. 4:**
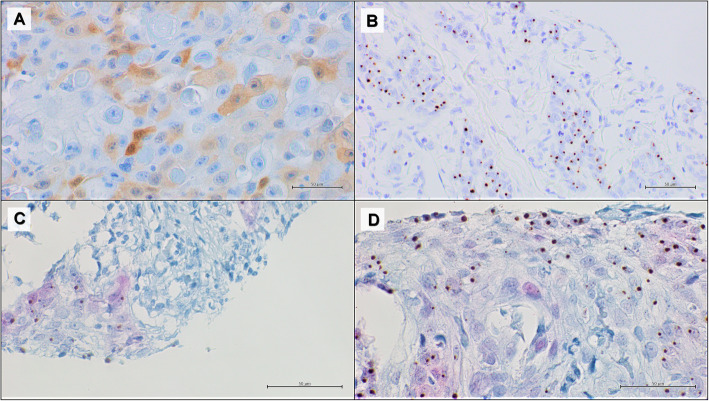
Representative results of an oropharyngeal squamous cell carcinoma with low p16 and HPV RNA expression. **a** positive p16 IHC, DAB staining (50x); **b** positive HPV RNA in situ hybridization, DAB staining (50x); **c-d** multiplex HPV RNA ISH/p16 IHC: positive p16 IHC Fast Red staining and positive HPV RNA ISH DAB staining (50x)

The multiplex HPV RNA ISH/p16 IHC results showed 100% concordance with the previous results achieved through the classic p16 IHC and HPV RNAscope carried out on two different slides (Fig. 3 and 4). The concordance of results obtained from the multiplex HPV RNA ISH/ p16 IHC with the classic IHC and ISH tests was blindly assessed from three different observers.

## Discussion

Detection of HPV-DNA can be performed through PCR or ISH, while the detection of HPV E6/E7 mRNA is based on ISH assay, and finally, p16 IHC represents an indirect method to establish a possible infection with the virus integration. DNA ISH has limited sensibility, mainly for tumor samples with low HPV copy numbers resulting in false negative results [23]. Moreover, the high possibility of cross-contamination associated with PCR-based HPV-DNA detection can lead to false-positive results [12–14]. PCR-based detection of the HPV E6/E7 mRNA transcripts can distinguish the transcriptionally-active HPV-related carcinomas from HPV infections clinically insignificant [14, 24]. HPV RNA ISH showed higher sensitivity and specificity compared to DNA ISH [19, 23, 25, 26]. Indeed, RNA ISH showed positive results also in cases that were negative or equivocal for DNA ISH test [23]. These discordant results explainable since the testing for HPV E6/E7 transcripts detects the presence of the integrated and transcriptionally active virus.

p16 overexpression has been described in approximately 20% of metastatic skin and lung HPV unrelated SCCs without a real association with HR-HPV [27]. Previous studies showed a high correspondence between p16 IHC and the RNAscope HPV-test, however, even if only in a few cases, discordant results have been found [16, 22, 23, 25]. Despite different methods were developed to directly or indirectly identify HPV, none of them would seem to have absolute high specificity and sensitivity. The rationale behind the development of a technique that integrates two HPV detection methods into a single assay is the possibility of completely remove false -positive and -negative cases.

The multiplex HPV RNA ISH/p16 IHC assay could be a promising assay to use in our routine considering its many advantages. This method is able to identify in a single assay both the p16 overexpression and the viral transcripts resulting in an increase in sensitivity and specificity to detect clinically relevant HPV-infections. The main advantage of this method is the easy interpretation of the results by a pathologist, since it is fully interpretable in light field. Furthermore, multiplex HPV RNA ISH/p16 IHC is technically feasible in FFPE tissue sections) and cytological samples, thus wide spreadable for routine testing in several clinical laboratories. The complete automation of the multiplex HPV RNA ISH/p16 IHC allows the reduction of time-consuming and the reproducibility of the technique, reducing any operator-dependent bias.

The novel multiplex approach proposed in this study could have some limitations that may concern particularly the expertise in the interpretation of the results and the optimization of the technique on other automation systems. Unlike the immunohistochemistry which has wide diffusion in all laboratories and is easily interpretable, the ISH represents a not widespread technique and moreover not always easy to interpret by all observers. In this view, this multiplex approach may be difficult to interpret for the observers with little expertise in the analysis of the in situ hybridization. In terms of the automation, although we have optimized this method on plataform Bond Leica, not too many technical difficulties are conceivable in the translation of our multiplex HPV RNA ISH/p16 IHC on other automatic platforms.

In the cervical carcinomas the HPV diagnosis is currently performed using the DNA-based molecular, rather than HPV RNA ISH [8]. HPV RNA ISH could have a role in resolving a differential diagnosis between the cervical low-grade squamous intraepithelial lesions (LSIL) and the reactive lesions, since p16 IHC showed low sensitivity and specificity in this context [28, 29]. Previous results in a series of cervical biopsies originally diagnosed as LSIL or low-grade neoplasia (CIN 1) showed that the detection of high- and low- risk HPV using HPV RNA ISH had 95% sensitivity and 98% specificity for cases diagnosed as CIN1 [29] Our novel approach that integrates p16 IHC and HPV RNA ISH analysis could represent a potential tool to improve the accuracy of LSIL/CIN1 diagnosis for morphologically ambiguous cases.

According to the current clinical practice, the multiplex HPV RNA ISH/p16 IHC approach could improve the identification of HPV-related SCCs. In head and neck cancer, HPV-SCC patients overall have significantly improved outcomes when compared to HPV-negative [30, 31]. HPV testing has clinical importance for the accurate identification of HPV-related head and neck cancer, not only prognostically, but also in treatment planning and follow-up [22].

The 8th edition of the cancer staging manual defined HPV-related OPSCC as a distinct entity, with staging now completely dependent on testing for p16 as a surrogate of HPV status [32]. in the clinical practise, approximately 8–20% of cases p16-positive OPSCC are HPV-negatives, suggesting the need for a more careful detection of HR-HPV [22, 33–35]. RNA ISH has considerably improved the HPV-positive OPSCC patient stratification, since its performance is incredibly high on FFPE samples and even on cytology cell blocks [19, 23, 27].

**Fig. 5:**
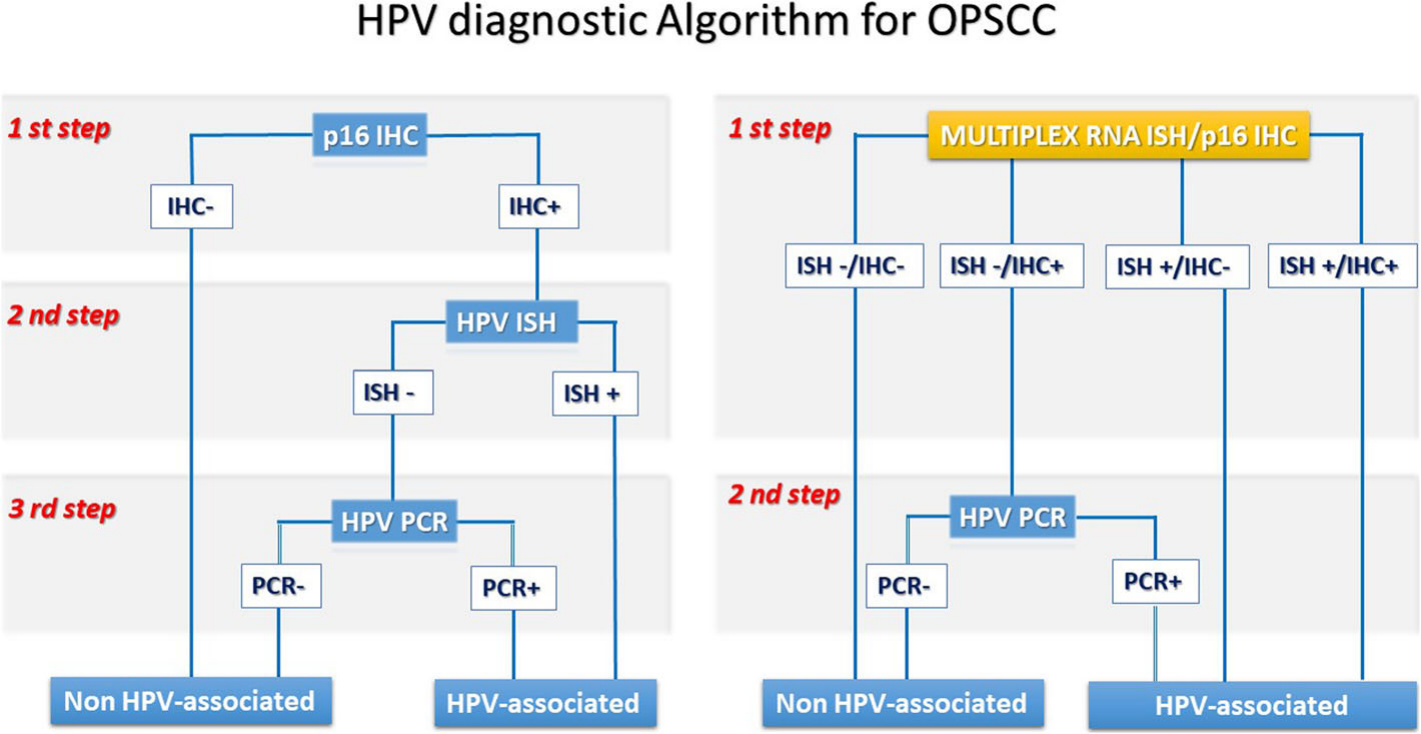
HPV diagnostic alghoritm for oropharyngeal squamous cell carcinoma OPSCC) including the multiplex HPV RNA ISH/p16 IHC assay

Previous authors proposed HPV diagnostic algorithms for OPSCC using the p16-IHC as the first stepwise to define tumors HPV-unrelated based on exclusively p16-IHC negative. Conversely, p16-positive potentially HPV-driven OPSCC are addressed to second molecular assays, namely ISH or PCR, to confirm the presence of HR-HPV [13, 36–38]. To date, the combination of p16 IHC and HPV RNA-ISH is necessary to increase the specificity required to avoid possible undertreatment of the HPV-related OPSCC patients with misdiagnosis of HPV-status [39]. Our multiplex approach could simultaneously provide two informations both the p16 expression and the HPV E6/E7 transcripts, resulting in a decrease of p16 false-negative OPSCC cases (Fig. 5).

Moreover, we hypothesize a further application field of our technique on cytological samples, particularly in OPSCC patients at an advanced stage when the cytology specimens are the only sample available to establish HPV-status [39, 40].

## Conclusions

Our results suggesting that the multiplex HPV RNA ISH/p16 IHC have several advantages, namely the feasibility in FFPE samples, the complete automation and a potential wide spreadable for clinical practice in several Pathology Unit. This novel approach could help to completely eliminate the percentage of misdiagnosed HPV-related cases saving time, costs and biomaterials.

## Data Availability

The datasets analysed during the current study are available from the corresponding author on reasonable request.

## Abbreviations

HPV: Human papillomavirus
HR-HPV: High-risk HPV
USPSTF: US Preventative Services Task Force
OPSCC: Oropharyngeal squamous cell carcinoma
PCR: Polymerase chain reaction
ISH: In situ hybridization
IHC: Immunohistochemisty
FDA: Food and Drug Administration
FFPE: Formalin-fixed paraffin-embedded
DAB: Diaminobenzidine
